# The clinical effects of antibiotic prophylaxis for hysteroscopic procedures: A meta-analysis

**DOI:** 10.1097/MD.0000000000016964

**Published:** 2019-08-23

**Authors:** Tao Guo, Ni Zeng, Jian Yang, Ping Wu, Pengpeng Liu, Zhisu Liu, Jun Cao

**Affiliations:** a Department of Hepatobiliary and Pancreatic Surgery, Zhongnan Hospital of Wuhan University, Wuhan; b Department of Reproductive Medicine Center, Hubei Maternal and Child Health Hospital; c School of Nursing, Huanggang Polytechnic College, Huanggang, PR China.

**Keywords:** antibiotic prophylaxis, hysteroscopic procedure, meta-analysis

## Abstract

**Background:**

Hysteroscopic procedures were widely applied but the clinical effects of antibiotic prophylaxis for these operations were not specifically addressed. In current study, we aimed to investigate the role of prophylactic antibiotics in hysteroscopic procedures by meta-analysis.

**Methods:**

We conducted literature retrieval in electronic databases, including MEDLINE, EMBASE, and Cochrane Central, to identify relevant randomized controlled trials (RCTs) investigating the clinical effects of antibiotic prophylaxis for hysteroscopic procedures. The postoperative infection rate was selected for pooled estimation. The *I*^2^ index statistic was used to assess heterogeneity. Publication bias was evaluated using funnel plots and Egger test. Sensitivity analysis based on different subcategories was conducted to examine the stability of the main results.

**Results:**

Four RCTs including 2221 patients were identified for the final quantitative analysis. Pooled estimation indicated no significant difference in infection rate between the antibiotic prophylaxis group and control group (test for OR: *Z* = 0.50, *P* = .616; 95% CI: 0.987–1.008). Sensitivity analysis based on surgical procedure, antibiotic application, follow-up time and administration time revealed similar results.

**Conclusion:**

Based on current objective evidence, we conclude that antibiotic prophylaxis exhibits no clinical benefit for hysteroscopic procedures. Therefore, it is not recommended. Meanwhile, more high-quality RCTs are needed to support our conclusion.

## 1 Introduction

Hysteroscopy is a safe, easy and successful one-session method for diagnosis and treatment of intrauterine abnormalities. It has become a widespread and accurate procedure for evaluating the uterine cavity because it offers direct visualization.^[1]^ It can be performed under sedation or local anesthesia, even on an outpatient basis, and it allows the examination to be less invasive and painful, thus reducing the need for inpatient services.^[2,3]^ Meanwhile, due to advances in endoscopic instruments and methods, reduced diameter of the instruments and less impairs provided more feasibility for hysteroscopic procedures including diagnosis and therapy.^[2,4]^ For instance, it is increasingly performed to screen infertile women for subtle intrauterine pathology in recent decades.^[4,5]^

On the other hand, clinical hysteroscopic procedures were mainly applied for diagnosis and therapy (such as diagnostic hysteroscopy, operative hysteroscopy and hystero resectoscopy). They may involve different operative processes, such as visually diagnostic checking, or with retrograde operations.^[2,3]^ Thus far, infection after hysteroscopy is uncommon, but its prevalence is estimated at approximately around 1% of cases.^[6,7]^ Thus antibiotic prophylaxis is not commonly considered to be a standard therapy, and its effects have not been specifically identified. However, postoperative infection complications are still a major concern in perioperative period because hysteroscopic procedures were performed in the relatively contaminated area, which has abundant bacterial flora, and the transcervical route may increase, per se, such a potential risk of local dysbacteriosis.^[8,9]^ In addition, hysteroscope insertion and removal may transfer vaginal and cervical flora into the uterine cavity. More importantly, a randomized controlled trial firstly reported by *Bhattacharya* claimed applying prophylactic antibiotics could significantly decrease the incidence of bacteremia for patients who underwent hysteroscopic surgery but revealed no clinical benefit for reducing essential infection rate.^[10]^ Thus, so far, the clinical value of antibiotic prophylaxis for hysteroscopy is not well defined, and there is no relevant guideline for prophylactic antibiotic standardization.

Given all these facts, it was necessary to conduct a quantitative, comprehensive comparison to elaborate the specific clinical efficacy of antibiotic prophylaxis for hysteroscopy. This study also aimed to provide clinical evidence for future standard guidelines.

## 2 Methods

### 2.1 Literature search and retrieval

Current meta-analysis was based entirely on previous published studies which had declared ethical approvals and no original clinical raw data was collected or utilized, thereby ethical approval was not conducted for this study. What's more, this study was conducted in strict accordance with the PRISMA guidelines,^[11]^ and this work has been registered online in PROSPERO (ID: CRD42019112622). To avoid local bias and to ensure the authority of the results, we conducted our literature retrieval only in globally recognized electronic databases, namely, MEDLINE, EMBASE, and Cochrane Central. Different combined mesh terms were applied to find precise studies addressing our target subject (an example of the search strategy in MEDLINE is presented in Table 1). The publication status and date were not restricted, but full English abstracts and texts were required.

**Table 1:**
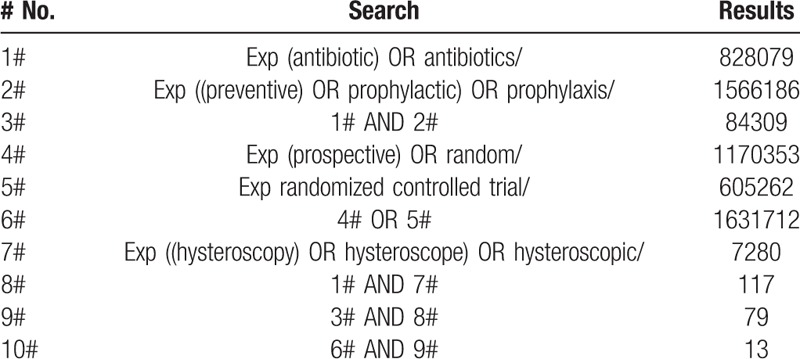
Example search strategy and process in MEDLINE.

### 2.2 Inclusion and exclusion criteria

The inclusion criteria were

(1) randomized controlled trials (RCTs) focused on hysteroscopic procedures;
(2) antibiotic prophylaxis was the only intervention between 2 arms;
(3) placebo, salines or no antibiotic treatment were regarded as control arm;
(4) due to improvements in surgical techniques, we only included trails performed after 2000.

The exclusion criteria were

(1) observational studies;
(2) no control group, or summarized experiences;
(3) reviews, comments, case reports or study protocols;
(4) trails on animal models;
(5) antibiotic prophylaxis was not the only intervention;
(6) trials performed before 2000;
(7) trials with insufficient raw data.

### 2.3 Raw data extraction and quality assessment

To evaluate the clinical effects of prophylactic antibiotics for hysteroscopic procedures, we planned to perform pooled estimation of infection rates from different RCTs. Therefore, raw data of infection rates were selected as the parametric data for meta-analysis. Infection was defined as any positive results of blood test or microbiological culture; symptoms (such as fever or pain) which could be cured by additional antibiotics; or clear diagnosis of relative postoperative endometritis, pelvic abscess, or cervicovaginitis.

Moreover, the included trials were assessed by the Cochrane Risk of Bias assessment tool^[12]^ to clarify the relative bias risk of each trial based on relative judgement bias terms of selection, performance, detection, attrition, reporting, and others. Relative graphics of bias risks for all included trials and the judgement for each trial were rated by Review Manager software (version 5.3).

The raw data extraction and bias risk assessment were independently conducted by 2 investigators. Any disagreements were resolved by group discussion with all team members.

### 2.4 Statistical analysis

In the present study, the infection rates were chosen for pooled estimation. Relative odds ratios (ORs) and associated 95% confidence intervals (CIs) were calculated. The statistical significance was set at *P* < .05. Moreover, the *Q* statistic was used to test for heterogeneity, and heterogeneity was considered significant if *P* < .05. Also, the *I*^2^ statistic was used to quantify heterogeneity with *I*^2^ > 50% implying substantial heterogeneity.^[13]^ Additionally, funnel plot symmetry and Egger test were used to assess the publication bias. Sensitivity analysis was conducted to assess the robustness of the main outcomes. Relative sensitivity and subgroup analyses were also performed among the following:

(1) surgical procedures;
(2) antibiotic application;
(3) follow-up time;
(4) administration time.

Subgroup data of pooled estimation and corresponding heterogeneity would be estimated. Data manipulation and statistical analyses of the meta-analysis were conducted using the Stata software package (version 12.0).

## 3 Results

### 3.1 Study characteristics and quality assessment

**Figure 1:**
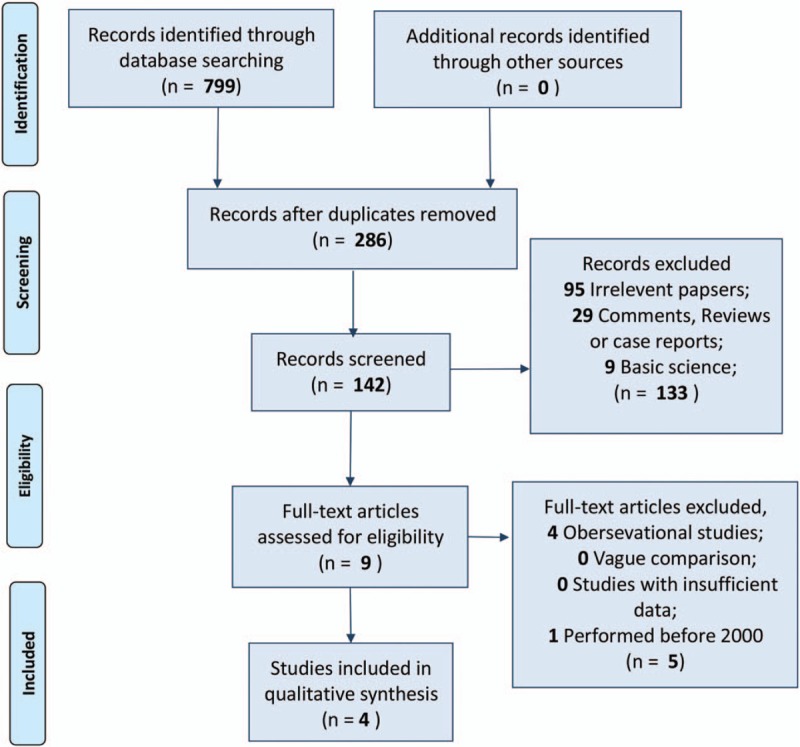
Flow diagram of the process of including and excluding studies for this meta-analysis.

**Figure 2:**
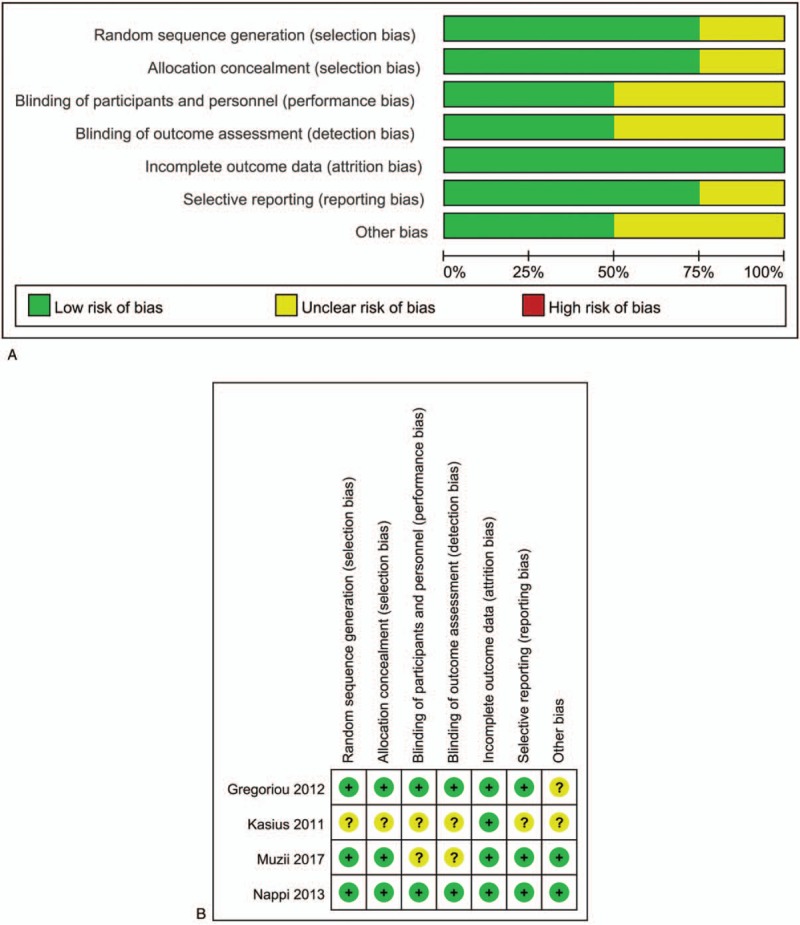
Bias assessment for included trials. (A) Risk-of-bias graph presented as percentages across all included studies. (B) Judgements regarding each risk-of-bias item for each included study.

After comprehensive electronic retrieval, 799 studies were searched, and 4 RCTs were finally identified for final quantitative estimation (Fig. 1). These 4 included trials^[14–17]^ contained 2221 patients, and all were performed in European countries (Table 2). On the other hand, for quality assessment, no obvious high-risk item was detected and 3 studies were designed with random sequence generation with concealed allocation, yet blinding was clear in only 2 trials (Fig. 2).

**Table 2:**
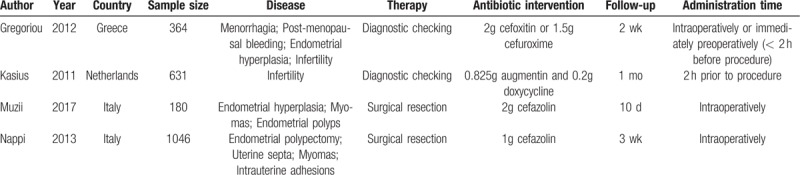
Characteristics of included studies.

### 3.2 Prophylactic antibiotics revealed no benefit for hysteroscopic procedures

**Figure 3:**
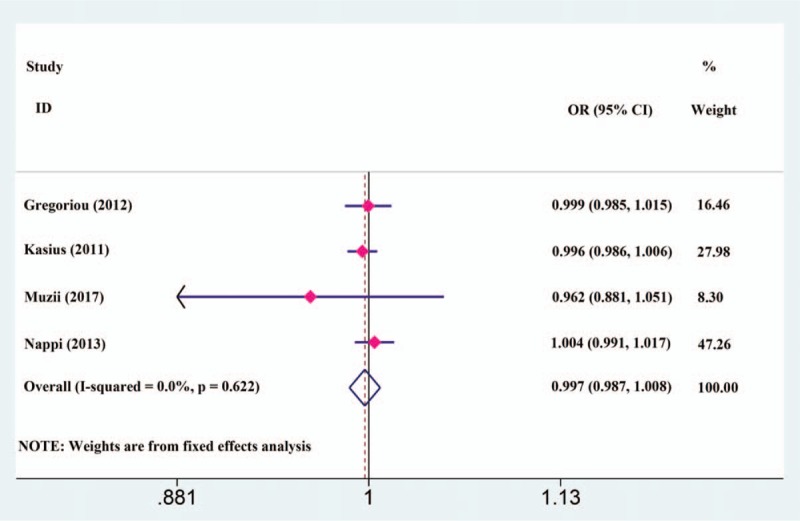
Forest plot between the antibiotic prophylaxis and control groups with respect to postoperative infection rates.

To investigate the clinical efficacy of antibiotic prophylaxis, we conducted quantitative pooled estimation of the infection rate. Based on fixed-effects modelling and with low heterogeneity (*I*^2^ = 0.00%), the results of the meta-analysis indicated no statistical significance between prophylactic antibiotics group and control group (test for OR: *Z* = 0.50, *P* = .616; 95% CI: 0.987–1.008) (Fig. 3). Given these statistical findings, we concluded that antibiotic prophylaxis brought no clinical benefit for patients who underwent hysteroscopic procedures.

### 3.3 Publication bias assessment

**Figure 4:**
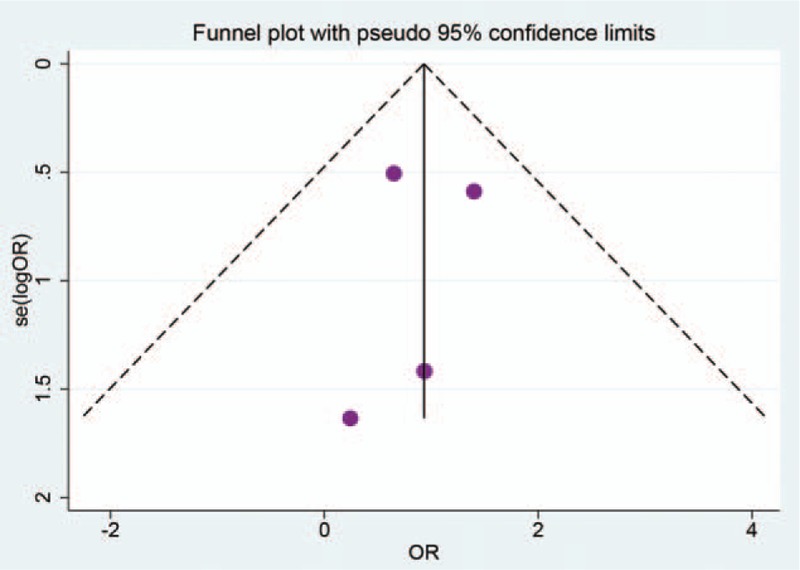
Bias evaluation based on funnel plot.

**Figure 5:**
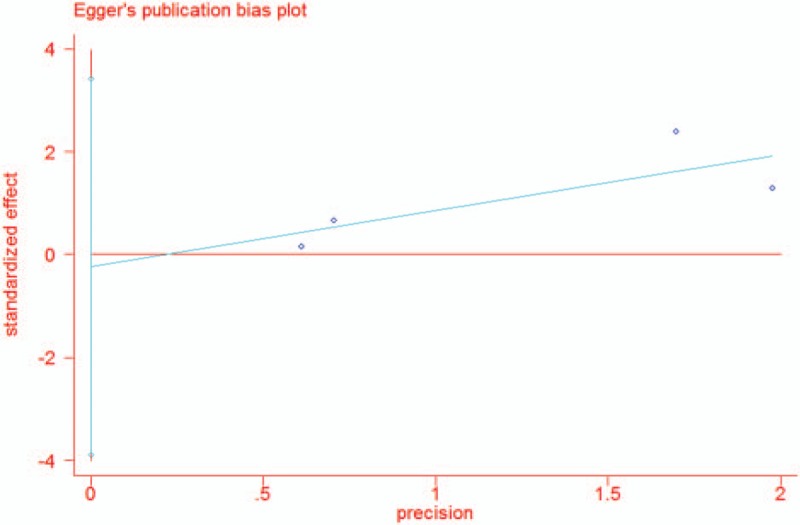
Egger publication bias plot regarding postoperative infection rate.

We measured publication bias using a funnel plot and Egger test. The effect size seemed to be concentrated and symmetrical in the funnel plot (Fig. 4), and the results of Egger test demonstrated that publication bias was highly improbable after statistical calculations (*P* = .807) (Fig. 5). According to these comprehensive quantitative evaluations, we concluded that no obvious publication bias existed in the assessed research.

### 3.4 Sensitivity analysis

We performed a sensitivity analysis to test the consistency between the subgroup analysis and the main results. First, we classified the included studies into 2 subgroups, namely, the diagnosis-only group and the surgical resection group, according to the surgical procedures. We discovered that both subgroup comparisons exhibited the same results (*P* = .509 and 0.780, respectively), which implied that prophylactic antibiotics had no efficacy for hysteroscopic procedures. We observed similar results in other subgroup analyses according to the other 3 subcategories (Table 3). Therefore, we demonstrated the consistency between the subgroup analysis and the main results. In addition, the sensitivity analysis showed the robustness of our main results.

**Table 3:**
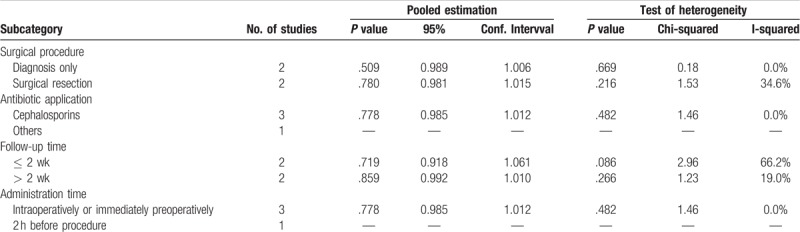
Results of sensitivity analysis regarding respective categories.

## 4 Discussion

The current meta-analysis was conducted with globally recognized electronic databases to ensure the authority of our results and to avoid local bias. We identified 4 RCTs from 3 European countries containing 2221 patients who underwent hysteroscopic procedures for our final quantitative pooled estimation. Based on fixed-effects modelling, our main results demonstrated that there was no significant difference between the antibiotic prophylaxis group and control group. The funnel plot symmetry and Egger test showed no obvious publication bias. Furthermore, a sensitivity analysis based on the subcategories of surgical procedures, antibiotic application, follow-up time and administration time exhibited similar results. Given all these findings, we believe that prophylactic antibiotics revealed no benefit for hysteroscopic procedures based on current evidence.

The clinical efficacy of antibiotic prophylaxis for operative hysteroscopy has been noticed since the last century,^[1,2]^ yet no quantitative estimation has been published. Prior to the current study, a Cochrane meta-analysis attempted to investigate the effect of antibiotic prophylaxis in women undergoing transcervical intrauterine procedures but failed due to the inability to identify any RCT.^[18]^ Moreover, 3 systematic reviews summarizing relative antibiotic prophylaxis for gynecologic procedures without quantitative analysis failed to provide clinical options and failed to reach consistent conclusions.^[19–21]^ Therefore, the current study was the first quantitative meta-analysis in this field, and we demonstrated that antibiotic prophylaxis is not necessary for hysteroscopic procedures. And all included trails were performed in the last decade which made the raw data meet the needs of today. Surgical procedures through or adjacent to the lower genital tract can have a moderate to high incidence of infection due to the abundance of bacterial flora. Therefore, antibiotic prophylaxis has been considered before gynecologic operations. And prophylactic antibiotic administration can reduce the incidence of infections in major gynecologic procedures, such as abdominal hysterectomy and caesarean section.^[22–24]^ However, our meta-analysis revealed that prophylactic antibiotic administration is not necessary for hysteroscopic procedures. These results may be attributable to the following factors. First, the relative hysteroscopic techniques and instruments have been improved, and the office hysteroscopic operation has reduced the distinction between outpatient and inpatient surgeries, shifting the focus in health care away from inpatient diagnosis and treatment. These developments have made this technique much easier, with shorter operative time than those done in the operating room. Second, the developed techniques have made it possible to miniaturize surgical instruments, which has further reduced the potential risk of infection.^[25]^ Most importantly, the acidic environment of the vagina forms a natural barrier against infection, and the vaginal-uterine surgical field keeps free communication with the outside. Thus, open and thoughtful condition theoretically reveals better postoperative drainage to reduce possibility of intraluminal infection, which is an essential difference from abdominal operations. For these reasons, the incidence of infectious complications after operative office hysteroscopic procedures is low and cannot be further reduced by prophylactic antibiotic administration.

Furthermore, sensitivity analysis revealed that different hysteroscopic procedures and antibiotic administrations have no impact on postoperative infections. This may indicate that no obvious independent factor influencing the infections was detected on current evidence. Notably, we noticed that no obvious high heterogeneity was detected in our study. We have mentioned that all included trails were conducted in European countries which have similar modern medical conditions. And in the last 2 decades, relative operative and aseptic techniques and instruments have achieved great promotion. These developments have made prophylactic antibiotics less important. Moreover, hysteroscopic procedures were applied in standard aseptic ways in Europe. These facts brought similar low infection rate and these also explained the low heterogeneity in current meta-analysis. On the other hand, antibiotic administration may cause side effects, the most severe being anaphylactic shock, which is rare but can be lethal.^[26]^ Meanwhile, the indiscriminate use of antibiotics has been associated with the development of antibiotic-resistant bacteria, and additional antibiotics may cost unnecessary medical expenses. Considering these drawbacks, we conclude that antibiotic prophylaxis is not recommended during hysteroscopic procedures as long as standardized aseptic procedures are performed.

Although this comprehensive meta-analysis provides objective evidence for the enrichment of clinical guidelines and decisions, we admit some shortcomings. First, the pooled estimation contained a large sample (2221 patients), and the funnel plot and Egger test demonstrated no obvious bias, but only 4 trials were included for quantitative analysis, and all were reported from Europe. These facts may have brought inevitable bias. Moreover, other details, which may also have impact on postoperative infections, such as operators’ techniques, modes of antibiotic administration, presurgical preparation, intraoperative exposure and irrigation liquids, could not be investigated at current stage due to inadequate raw data. Finally, according to our purposes and restrictive criteria, we might have omitted some studies and did not perform extensive enough analyses. Therefore, we believe that more relatively high-quality RCTs need to be conducted in the future.

In general, our final conclusion from this investigation is that antibiotic prophylaxis exhibits no benefit for hysteroscopic procedures and is not recommended for this procedure. More importantly, this study provides objective evidence for clinical guidelines and decisions. Meanwhile, more high-quality RCTs were needed to support our conclusions.

## Author contributions

**Conceptualization:** Tao Guo, Jun Cao.

**Data curation:** Tao Guo, Ping Wu.

**Formal analysis:** Tao Guo, Jian Yang.

**Investigation:** Tao Guo, Jian Yang, Pengpeng Liu.

**Methodology:** Tao Guo, Ni Zeng, Ping Wu, Pengpeng Liu.

**Project administration:** Zhisu Liu.

**Resources:** Ni Zeng, Jian Yang, Pengpeng Liu.

**Software:** Tao Guo, Ni Zeng, Jian Yang, Pengpeng Liu.

**Supervision:** Ni Zeng, Zhisu Liu, Jun Cao.

**Validation:** Ni Zeng, Jun Cao.

**Visualization:** Jun Cao.

**Writing – original draft:** Tao Guo, Zhisu Liu, Jun Cao.

**Writing – review & editing:** Zhisu Liu, Jun Cao.
